# A Case of De Novo Psoriasis Secondary to Nivolumab in a Patient With Metastatic Renal Cell Carcinoma

**DOI:** 10.7759/cureus.15703

**Published:** 2021-06-16

**Authors:** Sanjana Mullangi, Sreeja Ponnam, Manidhar Reddy Lekkala, Supriya Koya

**Affiliations:** 1 Internal Medicine, Hillcrest Medical Center, Tulsa, USA; 2 Neurosurgery, Oklahoma Surgical Hospital, Tulsa, USA; 3 Hematology and Oncology, University of Rochester Medical Center, Rochester, USA; 4 Hematology and Oncology, Hillcrest Medical Center, Tulsa, USA

**Keywords:** nivolumab, psoriasis, de novo psoriasis, renal cell carcinoma, pd-1 inhibitors, immune check-point inhibitor, drug-induced psoriasis, immune mediated side effects

## Abstract

Immune-mediated adverse events are commonly seen with immune checkpoint inhibitors like nivolumab. Oncology specialists usually have to screen patients for risk factors for autoimmune diseases, since immune checkpoint inhibitors can potentially exacerbate these events. Some of the immune-mediated side effects include polyneuropathies, colitis, and cutaneous adverse effects. Non-specific maculopapular rash, pruritus, lichenoid reactions, eczema, and vitiligo are the most common dermatologic side effects. It is thought that these adverse events are due to the blocking of the programmed cell death protein-1 (PD-1) pathway and are mediated by the cytotoxic T cells. Psoriasis has been previously reported as a side effect in a few case reports and most commonly presented as an exacerbation of preexisting psoriasis. However, de novo psoriasis occurrence as a result of nivolumab is a rare entity, especially in a non-melanoma patient. Here, we present a case of renal cell carcinoma treated with immunotherapy with nivolumab, who developed de novo psoriasis with palmoplantar involvement.

## Introduction

Programmed cell death protein 1 (PD-1) is considered to be an immune checkpoint molecule. Immune checkpoint inhibitors block the interaction between PD-1 and PD ligand-1, stimulating the T-cell activity and helping the anticancer host immune response [1]. Nivolumab is an IgG4 anti-PD-1 monoclonal antibody, which belongs to the class of immune checkpoint inhibitors and is approved in various cancers, including renal cell carcinoma [2]. The blockage of the PD-1 pathway is thought to cause immune-mediated adverse events such as organ-specific side effects and cutaneous side effects [3]. There are reports of anti-PD-1 induced de-novo psoriasis and exacerbation of previous psoriasis. De-novo psoriasis is usually seen in melanoma patients treated with anti-PD-1 inhibitors [4]. We report a case of a de-novo moderate to severe palmoplantar pustular psoriasis as a side effect from nivolumab in a renal cell carcinoma patient.

## Case presentation

**Figure 1:**
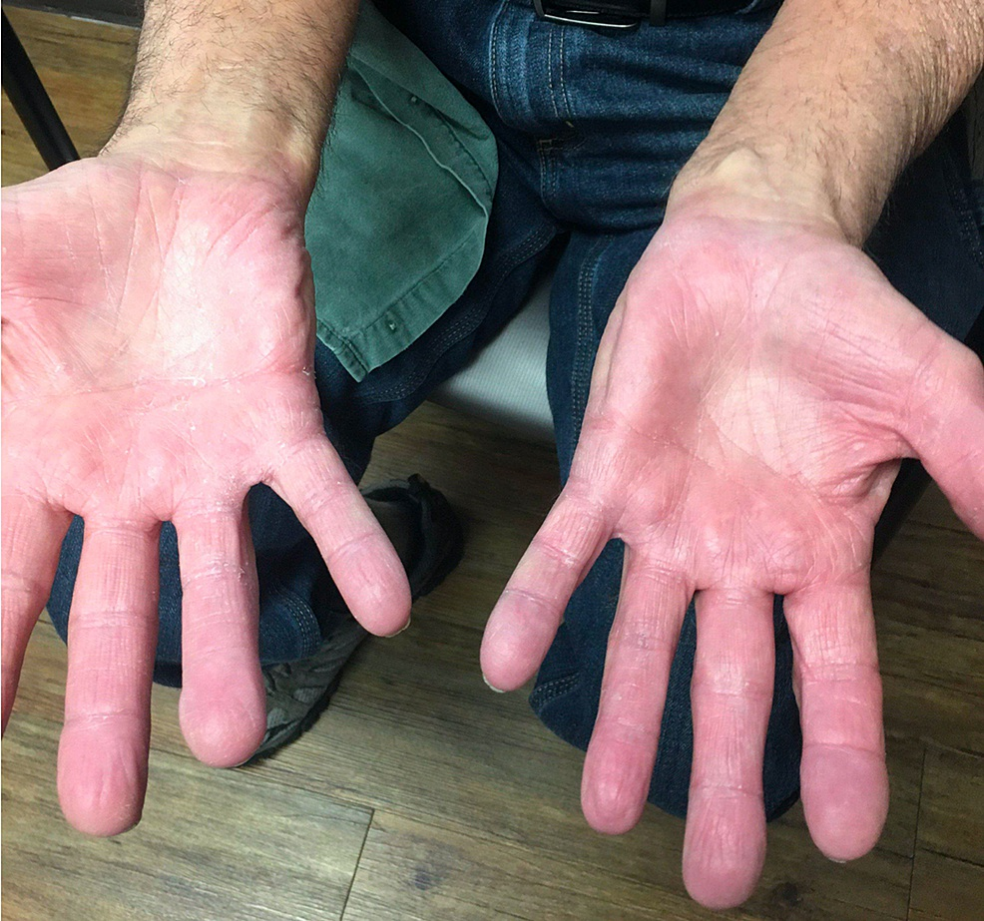
Showing improvement in the palmar psoriatic changes (Notice subtle symmetrical erythematous lesions with scaly plaques).

**Figure 2:**
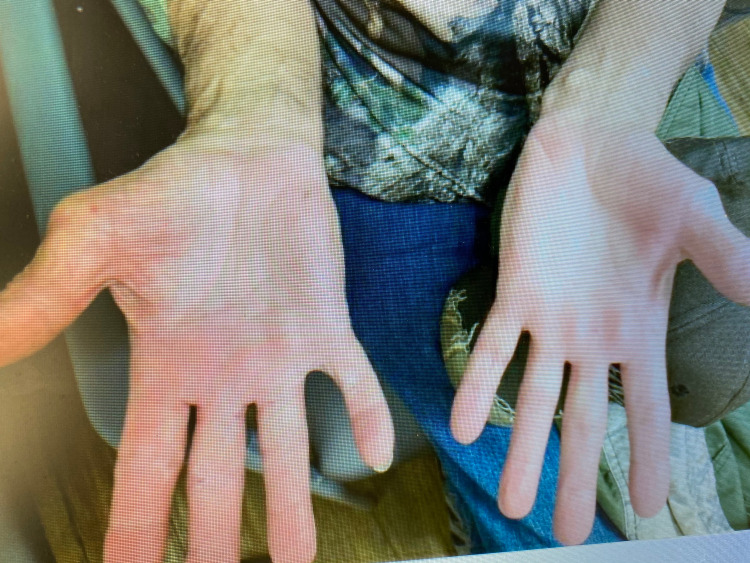
Notice further improvement from Figure 1, with almost baseline palmar surface.

A 66-year-old male with a past medical history of metastatic renal cell carcinoma presented with progressive rash while on nivolumab. He was initially diagnosed with Stage III renal cell carcinoma four years ago and underwent nephrectomy. Post-surgery, he was noted to have thyroid nodules on his scans, the biopsy of which indicated metastatic renal cell carcinoma. He underwent thyroidectomy and was started on pazopanib 800 mg daily two months after the surgery for his favorable risk renal cell carcinoma, which required dose adjustment due to side effects. A year later, he was noted to have a growing nodule in the right lower lung and underwent wedge resection. The biopsy showed a metastatic renal cell carcinoma. He was subsequently treated with axitinib and cabozantinib for the progression of the disease. However, he was switched to nivolumab seven months prior to presentation due to the progression of the disease. He tolerated the medication well before he developed progressive symptoms of rash on his palms, arms, and feet. He was evaluated by dermatology for his bright red, symmetrical, round, scaly, crusted, fleshy plaques and was diagnosed with palmoplantar psoriasis. He was started on topical steroids with triamcinolone, which didn’t help his symptoms, and needed apremilast and retinoids. Nivolumab was continued through these symptoms, but three months later, he developed severe diarrhea, requiring systemic steroids and infliximab. Nivolumab was held at the time. The repeat surveillance scans did not show any evidence of disease, and he continued to stay off therapy without recurrence of disease at his last follow-up two years after stopping nivolumab. Note the improvement noted in Figure 1 and Figure 2. 

## Discussion

Checkpoint inhibitors are being widely used, and their indications have been increasing. These medications are known to cause immune-mediated side effects and cutaneous toxicities frequently. Non-specific maculopapular rash, pruritus, lichenoid reactions, eczema, and vitiligo are the most common dermatologic side effects [5]. Psoriasis has been previously reported as a side effect in a few case reports and most commonly presented as an exacerbation of preexisting psoriasis [6]. The occurrence of de-novo psoriasis as a result of nivolumab is rare, especially in a non-melanoma patient but few cases were reported [7,8].

In patients with melanoma, the occurrence of psoriasis is thought to be due to the upregulation of metalloproteinase with thrombospondin motif-like 5 (ADAMTSL5) and disintegrin. The ADAMTSL5 specific CD8 + T cells are thought to trigger psoriasis, although this scenario is not applicable in non-melanotic cases [9]. It is known that PD-1 blockade augments the Th1 and Th17 responses, which correlate with the antitumor effect [10]. IL-17, the principal cytokine from Th17 cells, plays a key role in the pathogenesis of psoriasis. Furthermore, it is noted that PDL-1 is expressed in 95 percent of IL-17 producing T cells in psoriatic skin, which are activated by nivolumab [11].

There is a delay between the initiation of the anti-PD-1 and the development of psoriasis as seen in our patient. The delay is higher for de-novo psoriasis compared to exacerbation of underlying psoriasis [6]. Psoriasis is characterized by a wide range of clinical manifestations from mild to severe forms. Most of the patients exhibit asymptomatic, psoriasiform lesions, and immunotherapy can be continued in most cases. Psoriasis can be treated with vitamin D, topical steroids, oral steroids, and retinoids based on the severity of the lesions [5,6]. Less commonly, there are reports of palmoplantar involvement as was seen in our case.

## Conclusions

We report a rare case of de novo palmoplantar psoriasis occurring as a side effect of nivolumab treatment in a patient with metastatic renal cell carcinoma. Before initiating these immunotherapy drugs, patient history should be carefully checked for both family and personal immune-related diseases. Early recognition of side effects and prompt institution of appropriate immunosuppressive treatment can help continue checkpoint inhibitor therapy without interruption. 
